# Fabrication and characterization of electrospun PLLA/PANI/TSA fibers

**DOI:** 10.1039/c8ra10495f

**Published:** 2019-02-14

**Authors:** Junyan Yao, Yifu Chen, Wudan Li, Xiao Chen, Xiaodong Fan

**Affiliations:** School of Natural and Applied Sciences, Northwestern Polytechnical University Xi'an Shaanxi 710072 PR China; Key Laboratory of Macromolecular Science & Technology of Shaanxi Province, Northwestern Polytechnical University Xi'an Shaanxi 710072 PR China

## Abstract

Poly(l-lactic acid)/polyaniline/*p*-toluene sulfonic acid composites (PLLA/PANI/TSA) were synthesized *via* an emulsion polymerization-composition method with TSA as the dopant for PANI, and the structures and properties of the composites were characterized and analyzed. Conductive fiber tubes of PLLA/PANI/TSA were fabricated using electrospinning–knitting technology, and the effects of electrical stimulation on the growth and proliferation of osteoblast cells over the fiber tubes were investigated. The polymerization of PANI and the preparation of the PLLA/PANI/TSA composites were carried out *via* a one-step emulsion polymerization-composition or two-step method, where the reaction time of the two-step method was shorter and its yield higher than that of the one-step method. The addition of TSA improved the electrical conductivity of the PLLA/PANI composite and promoted chain propagation in the emulsion polymerization of the aniline oligomers and increased the degree of PANI polymerization. The PLLA/PANI/TSA fiber tubes obtained by electrospinning possessed regular morphologies and hydrophilic properties. Osteoblast cells grew and proliferated strongly with the PLLA/PANI/TSA fiber mats as a tissue scaffold and the electrical concentrating material under the condition of electrical stimulation of rectangular wave pulse signals with the appropriate stimulation current.

## Abstract 2

Poly(l-lactic acid)/polyaniline/TSA (PLLA/PANI/TSA) fiber mats play a positive role as a tissue scaffold for osteoblast cell proliferation.

## Graphical abstract

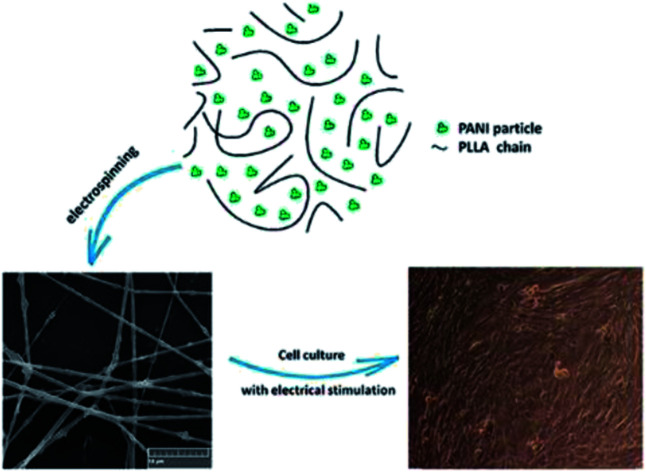

## Introduction

Conducting polymers are widely used in the optoelectronic field and have recently shown great potential in biomedical applications.^1–3^ For electroactive scaffold materials, conducting polymers can control the growth and proliferation of tissue cells by electrical stimulation.^4,5^ However, it is difficult for an exogenous electromagnetic field to achieve accurate localization.^6^ The loading electrical stimulus can be spatially regulated through conducting polymers by concentrating the electrical stimulation in the region of the polymer. Studies have shown that conducting polymer films such as PANI and polypyrrole films, as matrix materials, can support the adhesion, growth and proliferation of several cell types, including H9c2 cardiac myoblasts and PC12 cells.^7,8^ Among them, PANI has aroused great interest in recent years as a typical electroactive polymer due to its good environmental stability, low cost and unique doping mechanism.^9^ However, the biomedical application of PANI is still limited due to its inability to degrade, weak mechanical properties and poor processability.^10^ Herein, PLLA/PANI/TSA composites were synthesized by introducing PLLA and TSA as modified materials to improve the processability and conductivity of PANI, which were prepared as electroactive tissue engineering scaffold materials. The fibrous tissue engineering scaffolds are favorable for cell adhesion and proliferation due to their excellent three-dimensional reticulated structures, high porosity, high specific surface area and satisfactory biomechanical properties.^11^ In this study, PLLA/PANI/TSA electroactive fiber mats and tissue engineering scaffolds were prepared by electrospinning technology, and their structures, morphologies and electrical properties were also characterized. Furthermore, osteoblasts were cultured in the scaffolds and the influence of multivibrator signals on cell proliferation was investigated.

## Materials and methods

Poly(l-lactic acid) (PLLA, *M*_w_ = 13.2 × 10^4^ g mol^−1^) was purchased from American Nature Works Corporation. Aniline, chloroform, *N*,*N*-dimethylformamide (DMF), ammonium persulfate, sodium dodecyl sulfonate (SDS) and *p*-toluene sulfonic acid (TSA) were analytical reagents. Dulbecco's modified Eagle's medium (DMEM, 99%), fetal bovine serum (FBS, 99%), trypsin (>250 U mg^−1^) and thiazolyl blue (MTT, 98%) were purchased from Shanghai DaHao Biotechnology Co., Ltd.

### Preparation of PLLA/PANI/TSA fiber tubes and mats

PLLA/PANI/TSA2.4, PLLA/PANI/TSA4.1, PLLA/PANI/TSA5.3 and PLLA/PANI/TSA6.3 were synthesized *via* an emulsion polymerization-composition method, where the content of PANI was 15% and TSA was 2.4%, 4.1%, 5.3% and 6.3%, respectively. In a typical experiment, 0.177 g aniline, 0.103 g emulsifier (SDS) and a certain amount of TSA dissolved in 15 mL deionized water were added step by step into a 3-mouth flask in an ice–water bath with vigorous stirring. Subsequently, 0.65 g ammonium persulfate dissolved in 15 mL deionized water was dripped slowly into the flask. In the early stage of aniline polymerization or after polymerization for 4 h, 1 g PLLA dissolved in chloroform was dripped into the flask with vigorous stirring for 2 h. To remove the residual monomers, oligomer, ammonium persulfate and emulsifier, the products were washed with deionized water and ethanol until the water phase appeared colorless. The products in the oil phase were subjected to rotary evaporation and then dried under vacuum for 24 h to obtain the polymerized PLLA/PANI/TSA composites. Then the PLLA/PANI/TSA composites were dissolved and dispersed in a mixed solvent of chloroform/DMF (90/10, mass) using a magnetic stirrer and ultrasound device to prepare the spinning solutions of the PLLA/PANI/TSA composites (10 wt%). The solution was put into a syringe for electrospinning at the electrical voltage of 18 kV at the temperature of 25 °C. The distance between the syringe needle tip and the collector plate was 18 cm, and the feed rate was 12.7 μL min^−1^. The spinning speed of the collector was 15 m min^−1^, while the translational velocity was 10 mm min^−1^. The eletrospun fibers were treated in a vacuum oven for 24 h at 400 Pa and 40 °C to obtain the electrospun PLLA/PANI/TSA fiber tubes and mats. PLLA/PANI/TSA slice samples were processed by compression molding at 150 °C and 32 MPa.

### Characterization

Fourier transform infrared (FT-IR) spectra of PLLA/PANI/TSA composites were obtained using a Nicolet FT-IR spectrometer (Beijing Rayleigh Analytical Instrument Co., Ltd.). Scanning electron microscopy (SEM, Tescan) was used to observe the morphologies of the fiber samples. The SEM images were analyzed using the Image J software to determine the sizes of the fibers. The apparent density and porosity of the electrospun fiber mats were calculated using eqn (1) and (2) (ref. 12) as follows:1
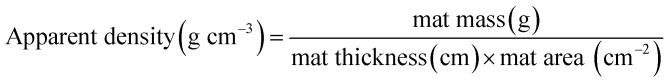
2
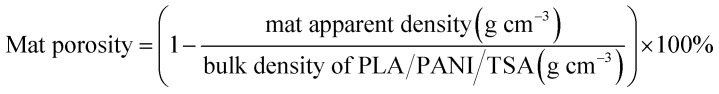
where, the thickness of the fiber mats was measured with a micrometer and the bulk densities of PLA/PANI/TSA2.4 and PLA/PANI/TSA5.3 were 1.274 g cm^−3^ and 1.272 g cm^−3^, respectively, as determined by the hydrostatic weighing method.

The resistivity of the PLLA/PANI/TSA slices was measured using an RTS-9 double electric four-probe meter. Water contact angles were measured using a JC2000D1 contact angle measurement instrument to characterize the hydrophilicity of the samples. X-ray photoelectron spectroscopy (XPS) was performed on the PLLA/PANI and PLLA/PANI/TSA composites using Kα radiation with a Kratos Axis Ultra DLD-α. The XPS Peak4.1 software was applied for peak separation and fitting.

### Cell culture and electrical stimulation

PLLA85/PANI15 (denoted as PLLA/PANI), PLLA/PANI/TSA2.4 and PLLA/PANI/TSA5.3 fiber tubes were cut to a length of 1.6 cm and then into rectangular fiber mats with even surfaces along each side. After sterilization under high temperature and high pressure for 1 h, the fiber mats were dried in an oven and sterilized with UV before cell culture. Mouse osteoblasts were seeded at a density of 2 × 10^5^ mL^−1^ on the PLLA/PANI, PLLA/PANI/TSA2.4 and PLLA/PANI/TSA5.3 fiber mats, and the culture plates were placed into an incubator filled with 5% CO_2_ at 37 °C. After seeding for 24 h, the electrical stimulation of the osteoblasts was carried out for 50 min every 24 h. The electrical stimulation signals were released by the device designed and assembled according to the principle of the 555 timer, which can output a series of current intensities of rectangular wave pulse signals according to the previous research.^13^ The stimulation current intensity was 9 μA, 18 μA and 50 μA, respectively, with a frequency of 50 Hz for a period of 500 ms. The positive and negative electrodes were inserted into the opposite ends of the fiber mats with a distance of 1 cm. After the 5th electrical stimulation, the osteoblast cells were taken out to investigate the cell proliferation using the MTT colorimetric assay.

### Design and assembly of the electrical stimulation device

The rectangular wave pulse signal was adopted as the electrical stimulation for the cell culture in the experiment. An electrical stimulation device that could generate rectangular wave pulse signals was designed and assembled with a 555 timer and related components. The value of resistance and capacitance in the circuit were determined using the formulas for frequency (*f*, eqn (3)) and duty ratio (*D*, eqn (4)).3
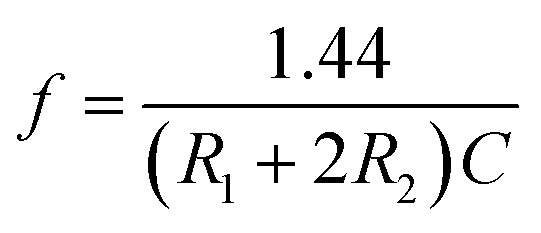
4
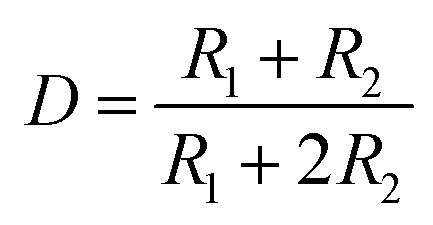


**Fig. 1:**
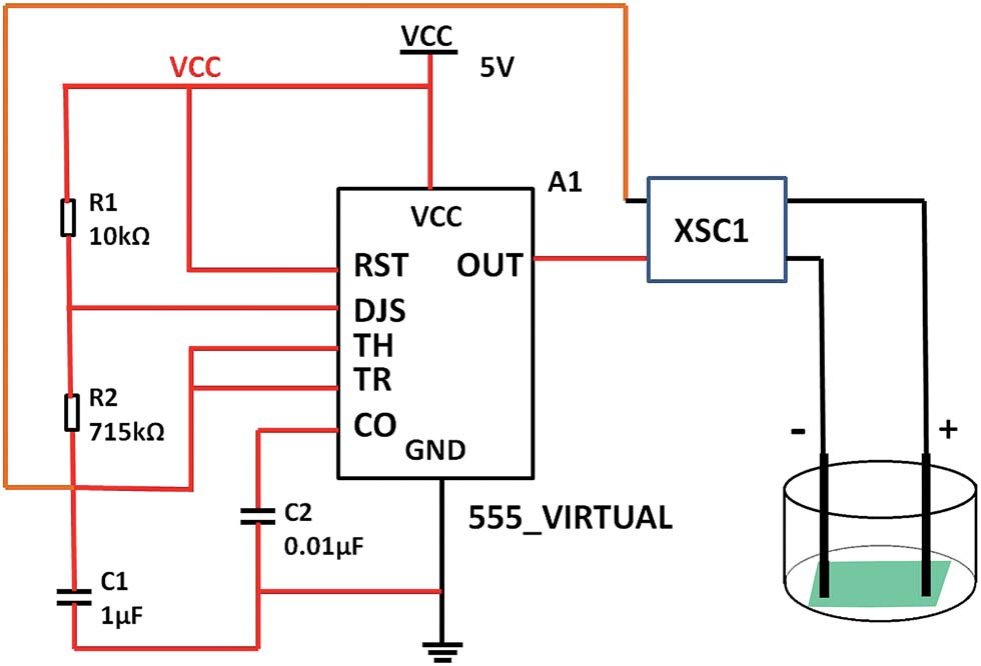
Schematic of the self-designed and assembled electrical stimulation device.

The 555 multivibrator circuit was simulated using the Multisim10.0 of circuit simulation software, and the electrical stimulation device was designed and assembled as shown in Fig. 1. To output rectangular wave pulse with an electrical signal of 1 Hz/500 ms, the resistors *R*_1_ and *R*_2_ were 10 kΩ and 715 kΩ, and the capacitors *C*_1_ and *C*_2_ were 1 μF and 0.01 μF according to the working principle of the 555 timer and eqn (1) and (2), respectively. While loading DC 5 V, the values of the other resistor was adjusted to keep the electrical stimulation 18 μA/1 Hz/500 ms (18 μA of current, 1 Hz of frequency, each cycle of stimulation lasted 500 ms). The electrodes contacted the fiber mat, and the distance between the positive and negative electrode was 1 cm.

## Results and discussion

### Comparison of the preparation of PLLA/PANI/TSA samples

It is difficult to prepare homogeneous PLLA/PANI/TSA composites by directly mixing the three ingredients due to the poor solubility of PANI, especially in the electrospinning solution.^14^ Thus, to improve the dispersion of PANI in PLLA, the PLLA/PANI composites were obtained *via* the emulsion polymerization method. Aniline emulsion was firstly polymerized into PANI particles for the PANI to be uniformly dispersed in the PLLA matrix.

In this study, the PLLA/PANI composites were prepared *via* a one-step or two-step emulsion polymerization-composition method. In the one-step process, aniline, emulsifier and water were added to a PLLA/chloroform solution successively. Then, the polymerization aniline occurred at the oil–water interface after an aqueous solution of oxidant was slowly added to the reactor. The total one-step reaction time was 18 h, with a yield 76.3%. In the two-step process, the PANI latex particles were synthesized *via* oil-in-water (o/w) emulsion polymerization for 4 h, and then the PLLA solution was added to a mixed solution and kept stirring for 2 h. The total two-step reaction time was 6 h, with a yield of 87.6%.

In the one-step process, the oil phase of aniline/PLLA/chloroform was firstly formed, and APS was dissolved in water to form the water phase because aniline is slightly soluble in water, while easily soluble in chloroform. Thus, an oil–water interface was formed. During the emulsion polymerization, the trace amount of aniline monomer dissolved in the water phase was initiated by APS to form a free radical with a short chain, which precipitated with an increase in the polymerization degree and was captured by the solubilization micelle. The polymerization continued and the short chains gathered into the micelles and the polymer nuclei germinated. Due to the chloroform diluting effect and steric hindrance of the PLLA chains, the diffusion speed of aniline from the oil phase to the water phase and the solubilization micelle was slow, leading to a decline in the polymerization rate and yield. The polymerization occurred on the oil–water interface, where the PANI particles gradually precipitated and dispersed into the PLLA solution.

**Scheme 1:**
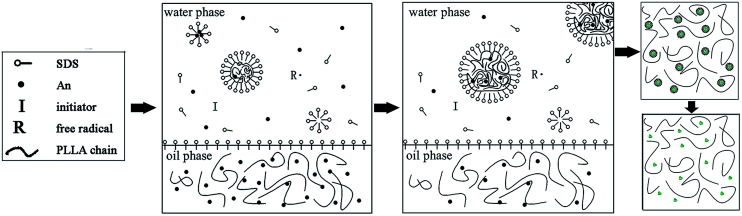
Schematic synthesis of PLLA/PANI by one-step emulsion polymerization-composition method.

**Scheme 2:**
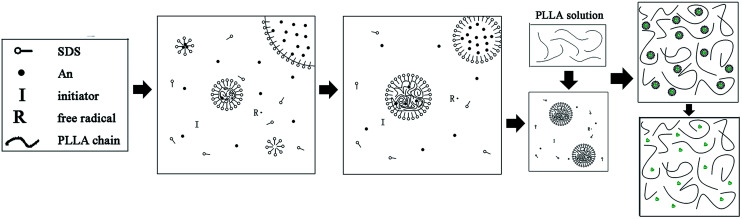
Schematic synthesis of PLLA/PANI by two-step emulsion polymerization-composition method.

In contrast, in the two-step method, the aniline monomer existed in the water phase and formed the droplets initially. The aniline polymerization was initiated by APS and generated free radicals with a short chain, which continued polymerizing to form PANI particles after being captured by the solubilization micelles. Then an emulsion was formed after the addition of PLLA/chloroform solution, thus the PLLA droplets and the PANI particles were dispersed uniformly. Without the dilution effect and steric hindrance during the emulsion polymerization, the aniline monomer could pass through the water phase quickly, enter the micelle and take part in the polymerization. Therefore, the reaction duration was shorter. Accordingly, we prepared the PLLA/PANI and PLLA/PANI/TSA composites using the two-step method due to its shorter reaction time and higher yield compared with the one-step method (Schemes 1 and 2).

### Structural conformation of the PLLA/PANI/TSA composites

**Fig. 2:**
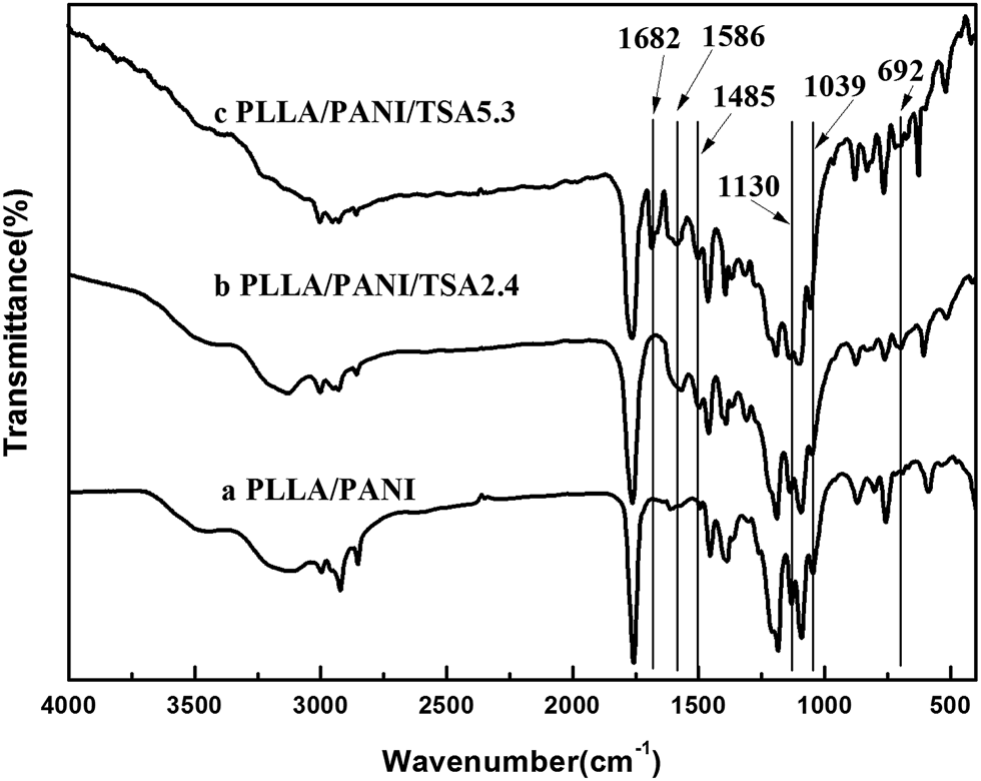
FT-IR spectra of PLLA/PANI, PLLA/PANI/TSA2.4 and PLLA/PANI/TSA5.3.

**Chart 1:**
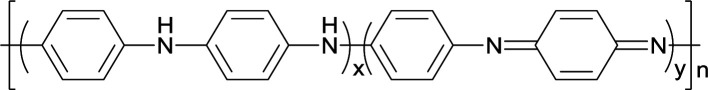
Chemical structure of PANI.

FT-IR spectroscopy was performed to characterize the structures of the main chain of the prepared PLLA/PANI, PLLA/PANI/TSA2.4 and PLLA/PANI/TSA5.3, and the results are shown in Fig. 2. Chart 1 shows the chemical structure of PANI, where, *n* is the polymerization degree, and *x* and *y* (*x* + *y* = 1) are the molar ratios of the two structural units.

As shown in Fig. 2, all the spectra of the composites showed strong bands at 1130 cm^−1^, 1039 cm^−1^ and 692 cm^−1^, which can be assigned to the stretching vibration of the quinoid rings of PANI, the C–O–C stretching vibration and the C–O in-plane bending vibration of PLLA, respectively. With the addition of TSA and increase in the TSA content in the composites, the absorption intensity of the three peaks was also enhanced, which may be due to the contribution of the anti-symmetrical and the symmetrical stretch vibration of S=O at 1130 cm^−1^ and 1039 cm^−1^, respectively, and the stretch vibration of C–S at 692 cm^−1^. In addition, all the TSA-doped composites showed the characteristic peaks for the conjugate vibration between the lone pair of electrons in the nitrogen atom on the PANI chains and the carbonyl group at 1682 cm^−1^, the quinoid ring at 1586 cm^−1^ and the benzene ring at 1485 cm^−1^. The FT-IR results indicated that TSA was successfully doped in the PLLA/PANI/TSA composites.

**Fig. 3:**
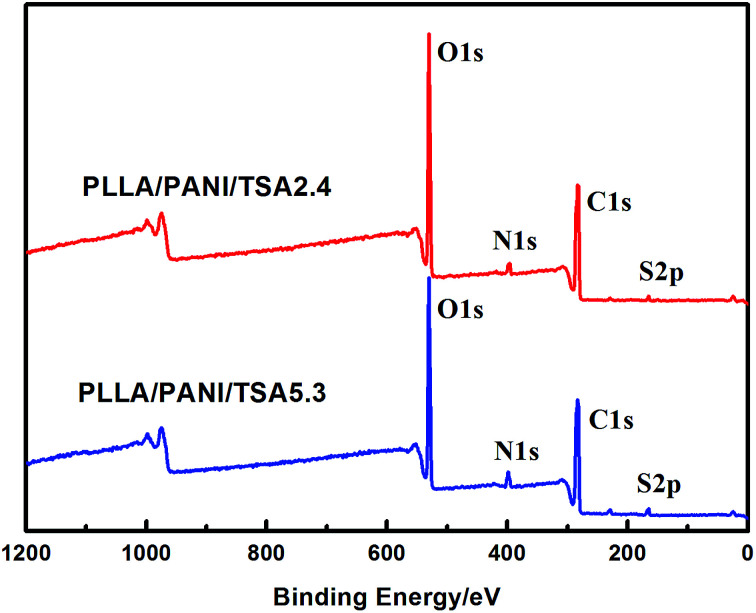
XPS spectra of PLLA/PANI/TSA2.4 and PLLA/PANI/TSA5.3.

**Fig. 4:**
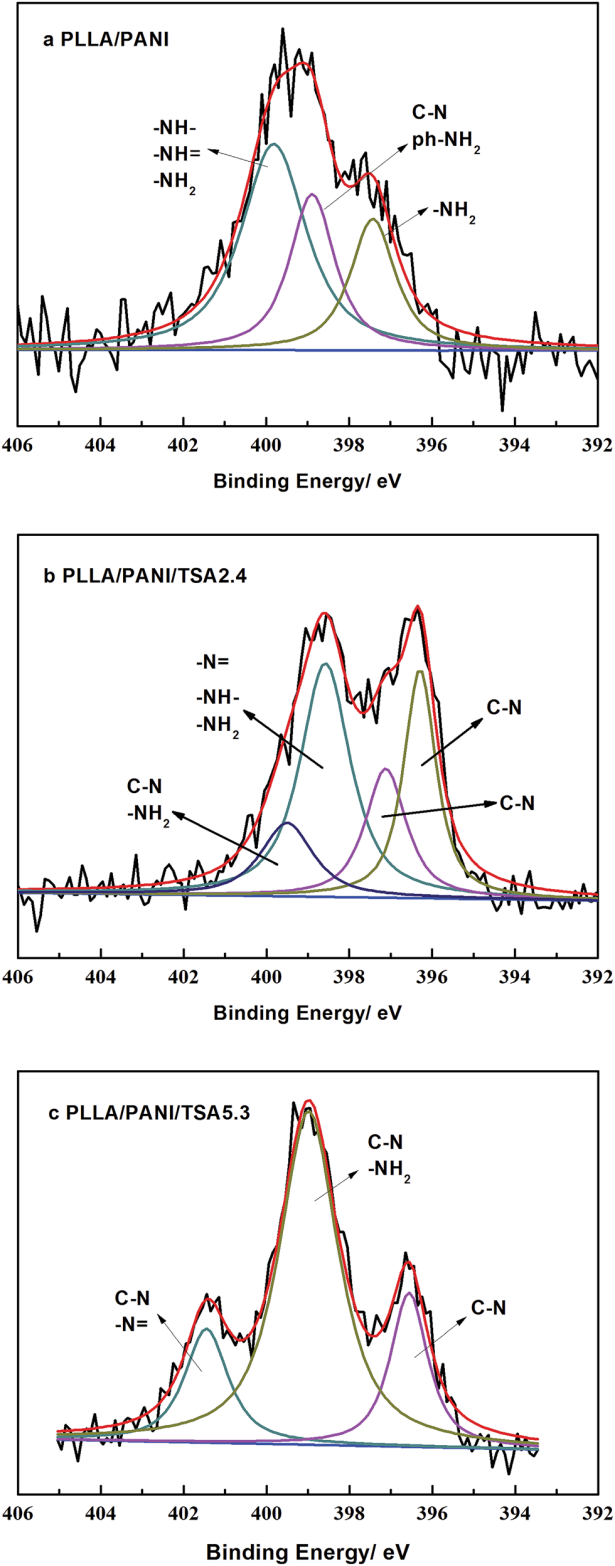
N 1s XPS peak-differentiating spectra of the composites. (a) PLLA/PANI, (b) PLLA/PANI/TSA2.4 and (c) PLLA/PANI/TSA5.3.

XPS analyses were performed to evaluate the chemical structures of the PLLA/PANI/TSA composites to study the polymerization of PANI deeply, and the results are shown in Fig. 3. The XPS patterns of the PLLA/PANI/TSA2.4 and PLLA/PANI/TSA5.3 composites displayed S 2p, C 1s, N 1s and O 1s peaks at binding energies of 165 eV, 283 eV, 399 eV and 530 eV, respectively. To investigate the connection ways between C and N, the peak separation and fitting of the N 1s spectra were conducted. The N 1s XPS-peak-differentiating analysis of PLLA/PANI/TSA2.4, PLLA/PANI/TSA5.3 and PLLA/PANI are given in Fig. 4.

There are four connection modes of nitrogen atoms in PANI, including –NH–, –NH_2_, =NH and –N=, where, –NH_2_ and =NH are the end groups of the PANI molecular chains, and –NH– and –N= are the connection bonds of the repeating units in the molecular chains. Based on the results of peak fitting, the binding energy peaks of PLLA/PANI N 1s could be deconvoluted into three parts, –NH_2_ (at 397.5 eV), –NH_2_ or C–N (at 399.0 eV), and –N=, –NH– or –NH_2_ (at 400.0 eV).

Additionally, the binding energy peaks of the nitrogen atoms of the PLLA/PANI/TSA2.4 could be deconvoluted into four species, C–N (396.3 eV), C–N (397.1 eV), –NH_2_, –N= or –NH– (398.6 eV), and C–N or –NH_2_ (399.5 eV). Obviously, the PANI molecular chains in PLLA/PANI/TSA2.4 contain a greater proportion of C–N repeating units, which means the polymerization degree of the PANI in the PLLA/PANI/TSA2.4 is higher than that in PLLA/PANI. In PLLA/PANI/TSA5.3, the binding energy peaks of the nitrogen atoms could be deconvoluted into three peaks, located at approximately 396.5 eV (C–N), 399.0 eV (–NH_2_ or C–N) and 401.5 eV (–N= or C–N). There are more end groups of PANI in PLLA/PANI than that in PLLA/PANI/TSA5.3, which indicates that the content of PANI oligomer in PLLA/PANI is higher. Specifically, the polymerization degree of PANI in PLLA/PANI/TSA5.3 is higher than that in PLLA/PANI.

D Nicolas-Debarnot^15^ reported that a certain acid environment can promote the propagating reaction of PANI. In our previous research, fullerene (C_60_) was doped into PANI as a conducting dopant, and consequently, the acidified C_60_ could promote the chain growth of aniline oligomers during the emulsion polymerization and improve the polymerization degree of polyaniline due to the carboxyl groups on the C_60_ surfaces. In this study, TSA, a strong organic acid, was doped into PANI as a conducting dopant. Meanwhile, the addition of TSA provided a favorable acid environment for the synthesis of PANI, which is beneficial for the head–tail connection of the cation radicals and the chain growth reaction. Thus, it effectively enhanced the chain growth reaction of PANI besides improving its electrical conductivity.

During the preparation of the PLA/PANI/TSA composite, TSA was dispersed more evenly in PANI, and its acid characteristics could play a more significant role in improving the polymerization degree of polyaniline than the acidified C_60_ because the TSA dissolved in the reaction system easily. Thus, the PANI molecular chains among the PLLA/PANI/TSA composites possessed higher molecular weights than that of the PLLA/PAN and PLLA/PAN/C_60_ composites.

### Conductivity characteristics of the PLLA/PANI/TSA composites

**Chart 2:**
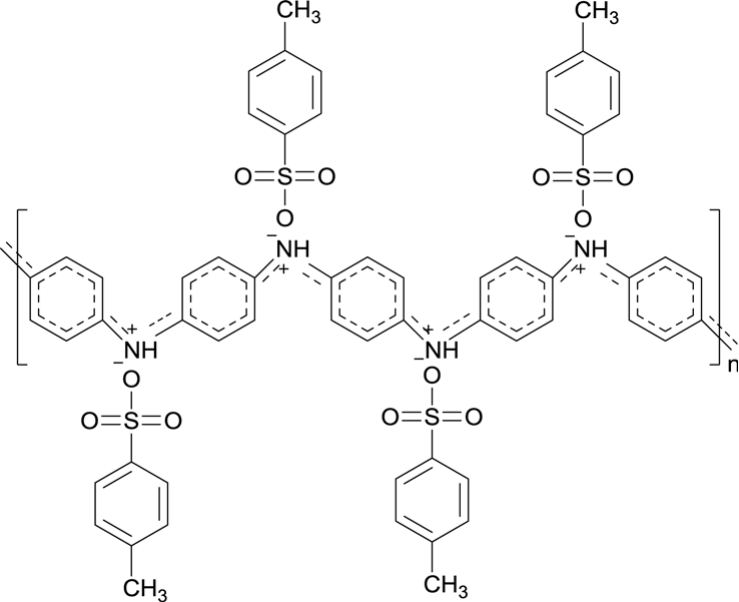
Molecular structure of TSA-doped PANI.

As an organic proton acid, TSA doping in PANI can increase the space of the PANI molecular chains and make them stretch, resulting in the promotion of charge delocalization and enhancement in the conductivity of PANI. The mechanism of TSA-doped PANI is illustrated in Chart 2.

**Fig. 5:**
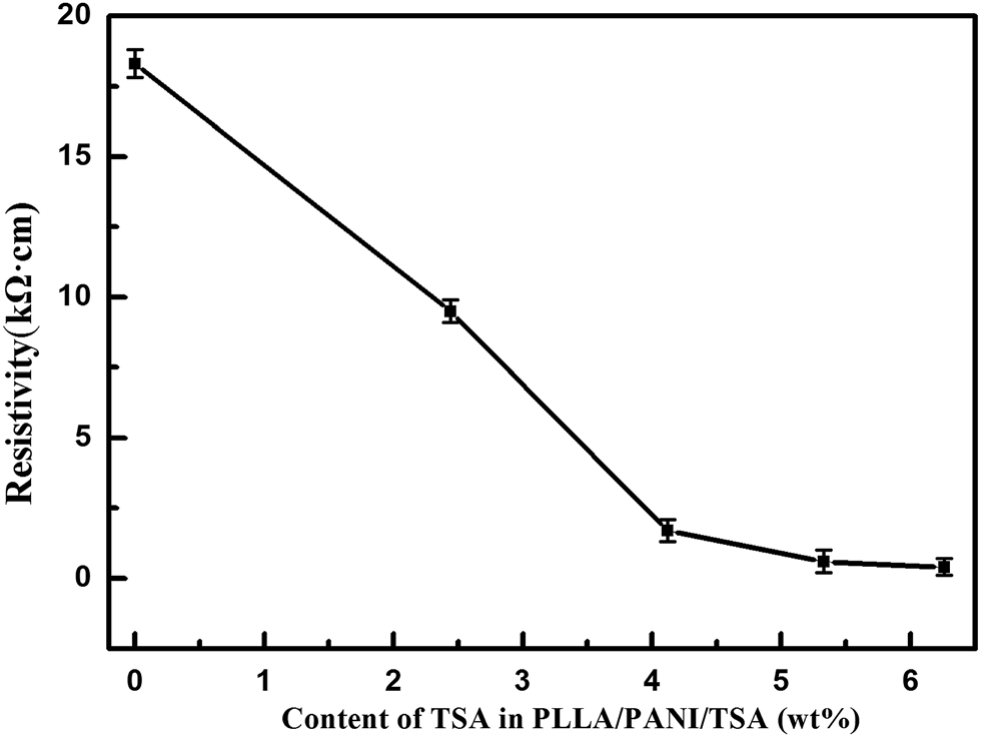
The resistance of PLLA/PANI/TSA doped with TSA.

The resistivity of the PLLA/PANI/TSA composites doped with various TSA concentrations was determined using a double electric four-probe meter (Fig. 5). As shown, the resistivity of the PLLA/PANI/TSA composites decreased with an increase in the TSA concentration. As a dopant in PANI, TSA is a non-oxidizing organic acid, which can promote the extension of the PANI molecular chains and the charge transformation due to the large pair of anions in its molecular structure. Therefore, the electrical conductivity of PLLA/PANI/TSA was greater than that of PLLA/PANI and PLLA/PANI/C_60_.^13^ When the mass content of TSA was 4.1%, the resistivity of the PLLA/PANI/TSA composites decreased rapidly. The declining trend became slower as the TSA content increase over 5.3 wt%. Nevertheless, an excess doping TSA dosage is inappropriate because excessive TSA, as an acid, may accelerate the degradation rate of PLLA in the composites, and consequently decrease the mechanical properties of the PLLA/PANI/TSA composites.

### Morphologies of the PLLA/PANI/TSA composites

**Fig. 6:**
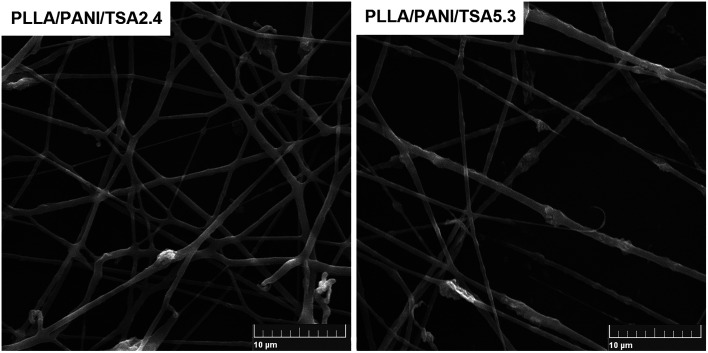
SEM micrographs of the PLLA/PANI/TSA fibers. Average diameters of PLLA/PANI/TSA2.4 fiber and PLLA/PANI/TSA5.3 fiber are 587 ± 164 nm and 529 ± 273 nm, respectively.

The SEM images of the PLLA/PANI/TSA2.4 and PLLA/PANI/TSA5.3 fibers fabricated *via* the electrospinning process are shown in Fig. 6. As shown, the PLLA/PANI/TSA2.4 fibers are randomly oriented with a smooth surface. Some fibers are flat rather than cylindrical and some are branched. This may be the result of the partial redissolution or bonding due to the incomplete evaporation of the solvent in under the conditions high air humidity or residual solvent, leading to collapse in the diameter direction. The diameters of the PLLA/PANI/TSA5.3 fibers are widely distributed, and the fiber surfaces are rough and apt to fracture, which indicate the fibers have poor toughness. This may be because the high content of TSA caused the TSA doping to fail and accelerates the degradation of PLLA in the fiber matrix. Consequently, the viscosity and surface tension of the composite electrospinning solutions decreased, and the entanglement effect between the molecular chains was weakened, leading to the fibers fracturing during spinning.

The receiving distance between the needle and the collector had a significant influence on the morphology and dimension of the electrospun PLLA/PANI/TSA nanofibers. The distance between the needle and the collector affects the tension and the flight time of the polymer solution jet flow in the electric field by changing the electric field strength. When the receiving distance is short, the electric field strength is increased, the jet velocity is increased, and the flight time is shortened. Thus, incomplete evaporation of the solvent occurs, and the fibers may appear partially fused. Partially interconnected fibers lead to an increase in the strength of the fiber mat, which is beneficial for the culture of cells in tissue engineering applications.^16,17^ Thus, partially interconnected fiber mats were prepared when the receiving distance between the needle and the collector was 18 cm, which is advantageous for cell culture.

In addition, the mixing uniformity of the composites in the spinning solution had an influence on the conductive pathway of the composites because the PLLA/PANI/TSA spinning solutions with two different doping proportions were conductive. Thus, the traction force of the electrospinning solution was affected, and the diameters of the PLLA/PANI/TSA fibers were changed. From the results of the analysis of the electrical performance of the PLLA/PANI/TSA composites, PLLA/PANI/TSA5.3 had better conductivity than the PLLA/PANI/TSA2.4 composites and the electrospinning solution. Therefore, the jet of the PLLA/PANI/TSA5.3 spinning solution was subjected to a larger traction force and orientation during the electrospinning process. The obtained fibers of PLLA/PANI/TSA5.3 were finer in diameter and easy to fracture. Consequently, the PLLA/PANI/TSA5.3 fiber exhibited a worse morphology in comparison to the PLLA/PANI/TSA2.4 fiber and its fiber diameter was also more inhomogeneous, which may induce further fracture and breakage.

During the electrospinning of the PLLA/PANI/TSA fibers, chloroform/DMF (90/10, mass) was chosen as the solvent for the spinning solution because DMF has a higher conductivity and has slower volatilization speed than chloroform. The nozzle was often clogged by the solidification of the PLLA/PANI/TSA solution when chloroform was used alone as the solvent because chloroform volatilizes rapidly. In comparison, the electrospinning PLLA/PANI/TSA solution seldom blocked the needle due to the lower volatilization speed of chloroform/DMF and the deposition rate of fibers increased,^18^ which was beneficial to the electrospinning process. In addition, the higher conductivity of DMF caused a stronger electric field force in the jet, which resulted in the axial whip of the jet, a decrease in fiber diameter and broadening of the fiber diameter distribution. The PANI/TSA in the solution significantly increased the conductivity of the spinning PLLA/PANI/TSA solution, which also caused a decrease in the fiber diameter. Therefore, chloroform/DMF (90/10, mass) was used as the solvent in the electrospinning process to prepare PLLA/PANI/TSA fibers.

However, an increase in the proportion of DMF in the chloroform/DMF compound solvent led to the slow evaporation of the electrospun solution. When the proportion of DMF in the chloroform/DMF compound solvent was over 12 wt%, the jet could not solidify in time after being deposited on the collector, which caused severe interconnection of the fibers. The normal morphology of the PLLA/PANI/TSA fibers could be obtained in the experiment where the proportion of DMF in the chloroform/DMF was in the range of 9 wt% to 12 wt% in the chloroform/DMF compound solvent.

When the spinning solution concentration was less than 6.5%, the spun product appeared as microspheres and beads. When the concentration of the spinning solution was higher than 12%, the needle was often clogged due to the rapid solidification of PLLA/PANI/TSA. Fibers with a regular morphology were obtained when the spinning solution had a concentration limited from 7% to 12%.

In our previous study, the average diameters of PLLA/bacitracin and the PLLA/mustard powder electrospun fibers were around 1634 nm (solvent dichloroethane/DMF, 90/10, mass) and 947 nm (solvent chloroform/DMF, 90/10, mass), respectively.^19,20^ The conductivities of the bacitracin and mustard powder were lower than that of PANI/TSA. The electrospun PLLA/PANI/TSA fibers had an average diameter of 500–600 nm, which indicates that an increase in the conductivity of the spinning solution reduces the average diameter of the fibers effectively.

The SEM photographs of the electrospun PLLA/PANI/TSA fibers showed the fibers were fused and dense. The partial interconnection among the fibers was considered as an advantage for the cultivation of cells in tissue engineering applications because the partial interconnection improves the strength of the fiber mats.^12,16^

The reasons for the partial interconnection of the fibers included the low evaporation speed of the chloroform/DMF compound solvent, the high conductivity of the electrospinning solution containing DMF and PANI, the high concentration of the spinning solution and the short receiving distance.

These factors accelerated the fiber deposition, reduced the flight time of the jet, and extended the solidification time. As a result, we obtained partial interconnected fibrous mats, which were propitious to the subsequent cell culture.

It has been reported that completely polymerized PANI has good biocompatibility, but is difficult to degrade.^15,21^ Generally, non-biodegradable materials that have fulfilled their duties *in vivo* need to be taken out *via* a second operation since they may cause a possible toxic effect. However, foreign particles with small diameters can be discharged by phagocytes or macrophages^21^ rather than *via* the two surgeries.

To date, there has been no report on the cell response to PANI. In this work, the prepared PLLA/PANI/TSA fiber mats were used for cell culture with electrical stimulation *in vivo*. The biocompatibility and the degradability are of interest because PANI, as foreign particles, was left *in vivo* after the cell culture and degradation and dissolution of the PLLA matrix. The PANI particles are supposed to remain stable in body fluid and not induce inflammation and infection due to their good biocompatibility. As shown in Fig. 6, the average diameter of the PANI particles was about 2.5 μm, and the maximum particle size was 3.1 μm. Thus, it can be concluded that the PANI particles can be phagocytized by phagocytes or macrophages and will not exist inside the body for a long period.

**Fig. 7:**
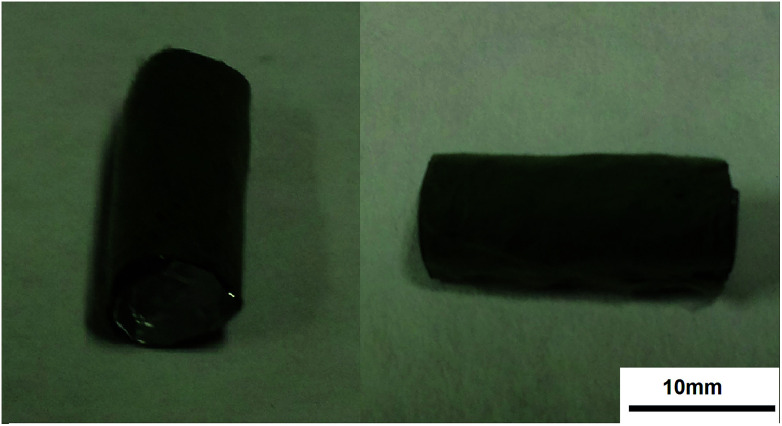
Photos of PLLA/PANI/TSA2.4 fiber tube.

Fig. 7 shows the fiber tube fabricated using the PLLA/PANI/TSA2.4 composite electrospinning solution at a concentration of 10 wt% and the optimized electrospinning–braiding process. As shown, the fiber tube has regular morphology and can provided a microstress environment for tissue culture, which has potential application in the tissue engineering field.

The stacked pores in the fiber mats were formed by the gaps between the adjacent fibers; thus, the fiber mats exhibited a porous morphology, which is favorable for nutrient transport and cell adhesion. The apparent density and porosity of the electrospun fiber mats were used to characterize their performance. By measurement and calculation, the apparent density and porosity of the electrospun PLA/PANI/TSA2.4 fiber mats were 0.38 g cm^−3^ and 70.5%, and that of the electrospun PLA/PANI/TSA5.3 fiber mats were 0.36 g cm^−3^ and 72.0%, respectively. The porosity of the PLA/PANI/TSA5.3 fiber mats was greater than that of the PLA/PANI/TSA2.4 fiber mats because the average diameter of the PLA/PANI/TSA5.3 fibers was smaller and their stacked density was lower due to the higher conductivity of the PLA/PANI/TSA5.3 solvent.

### Hydrophilicity of the PLLA/PANI/TSA composites

The hydrophobicity of PLLA hinders the adhesion and proliferation of cells; thus, it is necessary to improve its hydrophilicity. Table 1 summarizes the contact angle results of the PLLA/PANI/TSA flakes with different contents prepared using PLLA, PANI and TSA. The PLLA/PANI composites exhibited better hydrophilicity than PLLA because the PANI molecular chains contain hydrophilic amino groups. With an increase in the doping proportion of TSA, the contact angle of flakes with water tended to decrease. This is because the hydrophilic sulfonic group of TSA can further improve the hydrophilicity of PLLA/PANI/TSA, which is favorable to promote the early adhesion ability of cells on the fiber mats.

**Table 1:** Water contact angles of PLLA/PANI/TSA flakes

Samples	Molar ratio (TSA)/*n* (An)	Water contact angle (°)
PLLA	—	76
PLLA/PANI	—	71.5
PLLA/PANI/TSA2.4	0.2/1	62.9
PLLA/PANI/TSA4.1	0.4/1	55.2
PLLA/PANI/TSA5.3	0.6/1	52.5
PLLA/PANI/TSA6.3	0.8/1	49.4

### Osteoblast cell culture of the PLLA/PANI/TSA fiber mats

The residual electrospinning solvent in the fibers should be completely removed because chloroform is toxic to cells; thus, the eletrospun fiber mats were treated in a vacuum oven for 24 h at 400 Pa and 40 °C to evaporate the residual solvent inside. Subsequently, the fiber mats achieved constant weights.

**Fig. 8:**
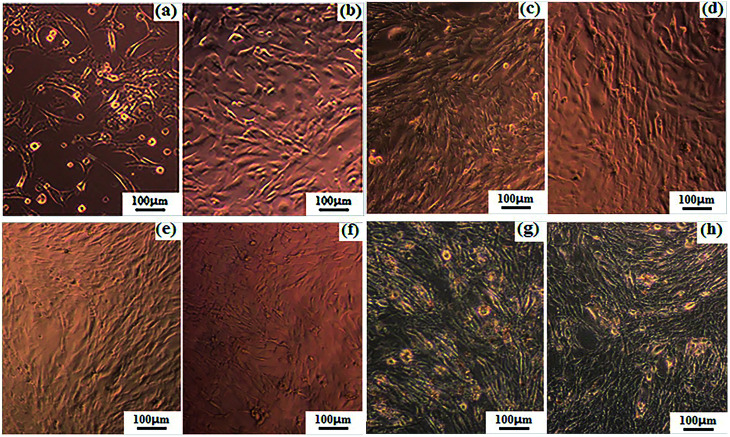
Microscopic images of the osteoblasts on (a)–(c) PLLA/PANI/TSA2.4 under electrical stimulation after 2, 4 and 6 days, respectively; (d) PLLA/PANI/TSA5.3 under electrical stimulation after 6 days; (e) and (f) PLLA/PANI without and with electrical stimulation after 6 days, respectively; and (g) and (h) blank group without and with electrical stimulation after 6 days, respectively.

Fig. 8 shows the microscopic images of the osteoblasts cultured both in the electrical stimulation groups (18 μA) and the electrical stimulation-free groups. As shown in Fig. 8(a)–(c), the cell densities of the osteoblasts adhered on the PLLA/PANI/TSA2.4 fiber mat gradually increased under the condition of electrical stimulation with an extension in the culture time. The cells spread well and were connected together with different shapes, such as short fusiform, triangle and irregular ellipse. Finally, the osteoblasts grew into a long fusiform shape and stretched out many pseudopodia. From Fig. 8(c), (d), (f) and (h), it could be observed that the cells cultured on both the PLLA/PANI/TSA fiber mats and in the blank group with electrical stimulation attached well, which secreted matrix and formed a network link. Furthermore, the cells grew successfully and distributed densely. Conversely, the growth of the cells on the PLLA/PANI fiber mat was not good and the spread of the cells were comparatively sparse. From Fig. 8(g) and (h), it can be inferred that there were more cells with electrical stimulation in the blank group and the cells were arranged more closely. However, the cells on the PLLA/PANI fiber mat with electrical stimulation had no significant increase in comparison with the electrical stimulation free cells (Fig. 8(e) and (f), respectively). Consequently, the cell proliferation with or without electrical stimulation is associated with the attachment materials.

**Fig. 9:**
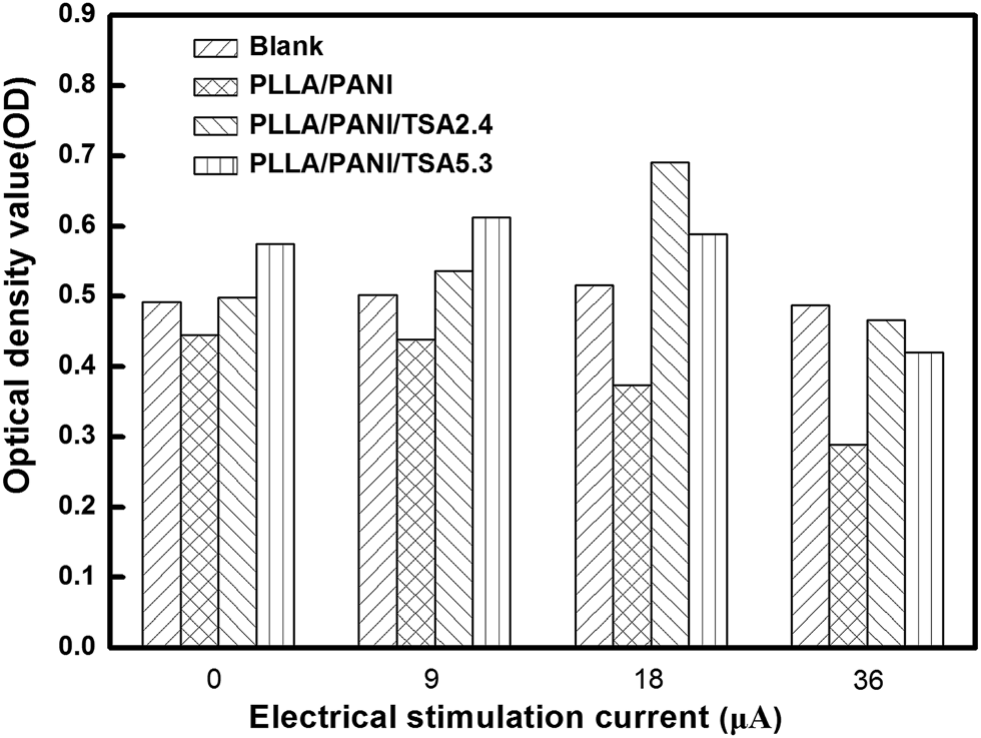
Optical density values (OD) of the osteoblast cells from MTT assay after 6 days culture.

Fig. 9 shows the optical density (OD) values of the osteoblasts from the MTT assays after 6 days culture. As shown in Fig. 8 and 9, when the electrical current was less than 18 μA, the number of cells on the two types of PLLA/PANI/TSA fiber mats was both higher than that in the blank groups. This is because the fiber mats have a high specific surface area and high porosity, which are conducive to adsorb water, protein and other nutrients, creating a favorable environment for cell attachment, and thereby promoting cell spreading and growth. However, when the current reached 36 μA, the number of cells on the two types of PLLA/PANI/TSA fiber mats was less than that of the blank group. The possible reason for the decline was that the higher electrical current stimulation may have promoted the release of the toxic substances in the fiber mats such as TSA or the PANI with a low molecular weight in the PLLA/PANI/TSA composites, which made it difficult for the osteoblasts to grow and proliferate. Since the acidity of TSA is beneficial to the high molecular weight of the synthesized PANI, the biocompatibility of the PLA/PANI/TSA fiber mats increased with a decrease in the poisonous effect on the osteoblast cells from the low molecular weight PANI in the composite, which resulted in a higher OD in the cell culture than that of PLA/PANI and PLA/PANI/C_60_ during electrical stimulation.

When the stimulation electrical current was 18 μA or 36 μA, the OD values of the cells on the PLLA/PANI/TSA2.4 fiber mats were higher than that on the PLLA/PANI/TSA5.3 fiber mats. This is owing to the high content of doping acid in PLLA/PANI/TSA5.3, which can accelerate the polymerization rate and increase the chance of chain termination, resulting in the synthesis of some lower molecular weight PANI. Meanwhile, the PLLA/PANI/TSA5.3 fiber mat doped with a high content of TSA possibly released some acid, which influenced the cell environment and was unfavorable for cell growth and proliferation. In contrast, when the electrical current was 9 μA, the OD value of the cells on the PLLA/PANI/TSA5.3 fiber mat was higher than that on the PLLA/PANI/TSA2.4 fiber mat, which was because the composition and internal structure of the PLLA/PANI/TSA5.3 fiber mat were relatively steady and low molecular weight substances were hardly released. Moreover, the PLLA/PANI/TSA5.3 fiber mat has better hydrophilic properties than the PLLA/PANI/TSA2.4 fiber mat. Hydrophilicity provides a good contact area for cell growth, which is beneficial to the early adhesion of osteoblasts on the fiber mats, increase in cell adhesion, and cell spreading and proliferation in the late stage.

The cells stimulated by the microcurrent of 18 μA grew well and proliferated sharply both in the blank control group and on the PLLA/PANI/TSA fiber mats with two doping proportions. Furthermore, the cell number increase in the electrical stimulation groups was more significant than that in the electrical stimulation-free groups. The cell OD values for the PLLA/PANI/TSA2.4 and PLLA/PANI/TSA5.3 fiber mats were higher than that in the corresponding electrical stimulation-free groups by 38.8% and 2.4%, respectively. The OD value of the cells in the blank group with the electrical stimulation was 4.9% higher than that with no electrical stimulation. These results suggest that the appropriate electrical stimulation is advantageous to early adhesion and spreading of the osteoblasts, and it can promote the growth and proliferation of osteoblasts. This is because the early adhesion and spreading of osteoblasts are important for their growth and proliferation.

From the above results and analysis, it can be concluded that the appropriate electrical stimulation current on fiber mats can accelerate cell growth and proliferation. The PLLA/PANI/TSA2.4 fiber mat was more beneficial for cell growth and proliferation than the PLLA/PANI/TSA5.3 fiber mat at the electrical stimulation current of 18 μA.

## Conclusions

Well-dispersed and biodegradable PLLA85/PANI15/TSA composites were synthesized *via* a two-step emulsion polymerization-composition method. PLLA/PANI/TSA fiber mats and fiber tubes were further fabricated *via* electrospinning–knitting. TSA, as a dopant in PANI, was doped successfully into the PLLA/PANI/TSA composites and enhanced the conductivity of PLLA/PANI. Also, the addition of TSA created an acid environment for the polymerization of aniline, which increased the molecular weight of PANI. The PLLA/PANI/TSA fibers and the fiber tubes had regular morphologies, which were stable and uniform, and thus could retain their structure and morphology during the tissue culture. Accordingly, the PLLA/PANI/TSA2.4 fiber mat was conductive and promoted the cell adhesion, growth and proliferation of osteoblasts cultured on it with the electrical stimulation of a rectangular pulse wave at 18 μA/1 Hz/500 ms.

## Conflicts of interest

There are no conflicts to declare.

## Supplementary Material
